# Autoimmune encephalitis with dizziness in children: A case report presented to the otolaryngology department

**DOI:** 10.1097/MD.0000000000041558

**Published:** 2025-02-14

**Authors:** Guifang Li, Anqi Zhang, Xinge Lu, Hua Liang, Jinglei Fang, Yabo Wang, Yanzhuo Zhang

**Affiliations:** a Department of Ophthalmology, Hebei Provincial Eye Hospital, Hebei Provincial Key Laboratory of Ophthalmology, Hebei Provincial Eye Institute, Xingtai, Hebei, China.

**Keywords:** autoimmune encephalitis, case report, dizziness, MOGAD, retrobulbar neuritis

## Abstract

**Rationale:**

Motion sickness is frequently encountered condition, characterized primarily by symptoms such as nausea, vomiting, headache, dizziness, and drowsiness. Autoimmune encephalitis refers to a group of diseases that can present with a variety of clinical symptoms according to the expressed autoantigen. One of the rare types is myelin oligodendrocyte glycoprotein antibody-associated disease (MOGAD). Herein, we report the case of a child who presented to our otolaryngology department with a diagnosis of motion sickness but was eventually diagnosed with MOGAD. To our knowledge, this is the first such case reported in the literature.

**Patient concerns:**

An 11-year-old boy presented to a vertigo clinic with the primary complaints of dizziness, occasional nausea, and vomiting after traveling with his family for 3 days. He was diagnosed with motion sickness and was administered oral betastine mesylate tablets (6 mg, 3 times/d for 3 days). The dizziness resolved after 3 days of medication. However, 1 week later, the child developed symptoms of vision loss, poor mental performance, and sluggish responses.

**Diagnosis:**

The patient was diagnosed with MOGAD.

**Interventions:**

The patient was treated with human immunoglobulin (2 g/kg, divided into 3 doses), mannitol, and a high dose of methylprednisolone succinate (20 mg/kg for 3 consecutive days). The dose of methylprednisolone succinate was then gradually tapered over 18 days.

**Outcomes:**

After 15 days of treatment, the child’s vision in the left eye improved, with visual acuity returned to 0.25. His sense of taste and pain in the left limb returned to normal, his mental responses were good, and no antinuclear antibodies were detectable.

**Lessons:**

Although most childhood episodes of dizziness are benign, it is important for clinicians to remain vigilant for the possibility of central nervous system disease as the underlying cause. When the diagnosis is uncertain, doctors and parents must closely monitor affected children to avoid misdiagnosis and treatment delays.

## 1. Introduction

Dizziness or vertigo is common in children who visit otolaryngology clinics; however, most cases are benign. The most common related disorders in this patient group are benign recurrent vertigo (BRV) and vestibular migraine (VM), migraine-related diseases that account for 40% of cases.^[1]^ Motion sickness, also known as travel sickness or seasickness, is another common cause of dizziness in children.^[2]^ Although previous studies have shown that motion sickness occurs in 67% of adults and 56% of children, these findings have not been established with statistical significance.^[3]^

Myelin oligodendrocyte glycoprotein (MOG) antibody-associated disease (MOGAD) is a category of central nervous system (CNS) demyelinating disorders caused by autoantibodies against MOG. This autoimmune disease primarily targets the optic nerve and spinal cord, leading to visual impairment and motor paralysis.^[4,5]^ MOGAD can affect individuals of any age. In children, it typically causes acute disseminated encephalomyelitis, while optic neuritis and myelitis are more common in adults.^[6,7]^

Herein, we report the case of a child who presented to our otolaryngology department with motion sickness. He later developed visual impairments and unresponsiveness, and was eventually diagnosed with MOGAD.

## 2. Case presentation

This study was approved by the Medical Ethics Committee of Hebei Provincial Eye Hospital. An 11-year-old boy presented to the hospital with primary complaints of dizziness, occasional nausea, and vomiting after traveling with his family for 3 days. His dizziness was persistent and initially accompanied by nausea and vomiting, but without headaches or disturbances of consciousness. These symptoms did not improve after 2 days of rest.

**Figure 1.**
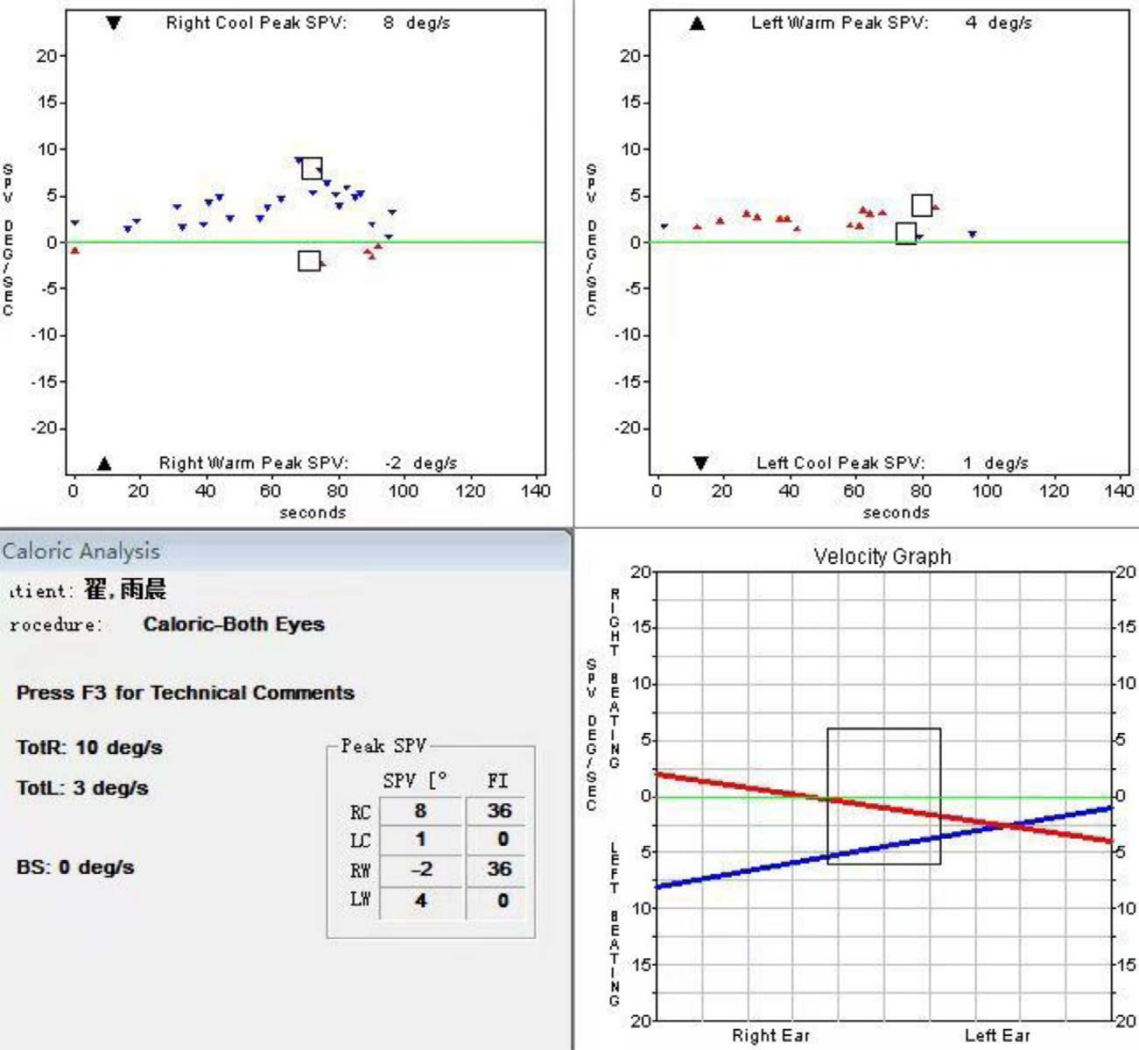
Bithermal caloric test at August 23, 2022. After the right ear was injected with cold air and hot air, the sum of slow phase velocity was 10°/s, while that of the left ear was 5°/s, and the canal paralysis value is 33%, which was >25%, indicating that the low-frequency response of the left horizontal semicircular canal was reduced.

Physical examination revealed no remarkable findings. A bithermal caloric test of vestibular function revealed a decreased low-frequency response in the right horizontal semicircular canal (Fig. 1). Given the patient’s previous history of motion sickness and an motion sickness susceptibility questionnaire short-form (MSSQ-short) score of 85%, he was diagnosed with motion sickness and administered oral betastine mesylate tablets (6 mg, 3 times/d for 3 days). His parents were advised to monitor for improvements or exacerbations of his condition and to return for a follow-up at any time.

The patient’s dizziness resolved after 3 days of medication; however, 1 week later, symptoms of vision loss, poor mental performance, and sluggish responses occurred. Physical examination revealed that his visual acuity was 0.1 in the left eye and 1.0 in the right eye, his sense of taste was reduced, and the pain reflex in his left lower limb was slower than that in the right. Fundoscopy revealed no congestion or edema in the optic disc, and magnetic resonance imaging (MRI) of the orbit showed normal results.

**Figure 2.**
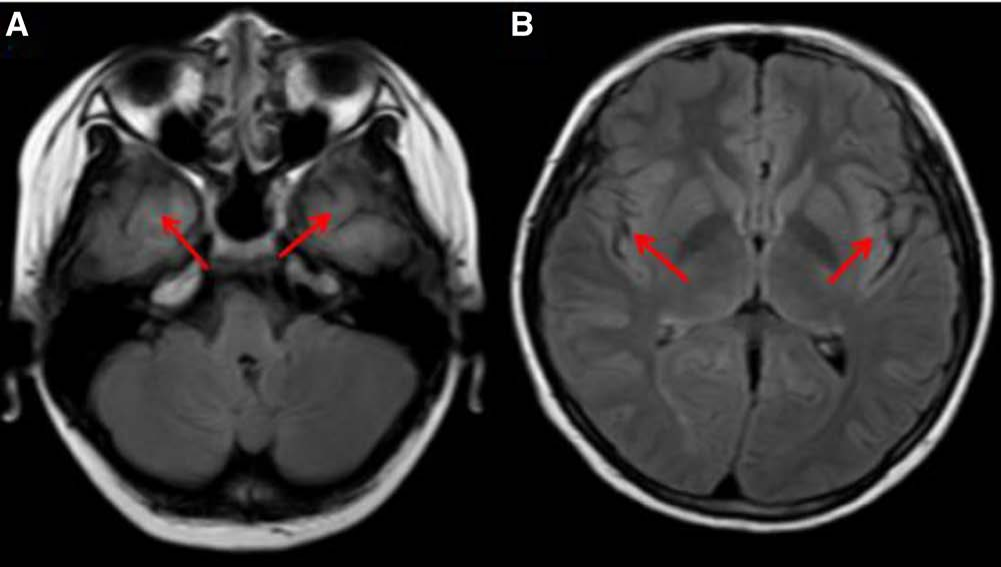
Cerebral MRI in FLAIR image at admission. (A) showed hyperintense lesion in the temporal regions (arrowheads). (B) showed hyperintense lesion in the bilateral insular regions (arrowheads). FLAIR = fluid attenuated inversion recovery, MRI = magnetic resonance imaging.

The patient was referred to the pediatric department and admitted for treatment. Cerebral MRI showed hyperintense lesions in the bilateral insular and temporal regions on T2-weighted images and hypointense lesions on T1 images, accompanied by hyperintensity on fluid attenuated inversion recovery (FLAIR) images (Fig. 2). Electroencephalography showed a mixed rhythm of 7 to 8 Hz with low- to medium-amplitude activity in the bilateral occipital region, as well as multiple low-amplitude fast waves. The left and right regions were mostly symmetrical during wakefulness and quietness when the patient’s eyes were closed, whereas the rhythm in the occipital region was suppressed during wakefulness with eyes open. His serum demyelinating autoimmune antibody test was positive for MOG-IgG (titer = 1:10), and a cerebrospinal fluid (CSF) oligoclonal band examination performed 3 days later was also positive.

**Figure 3.**
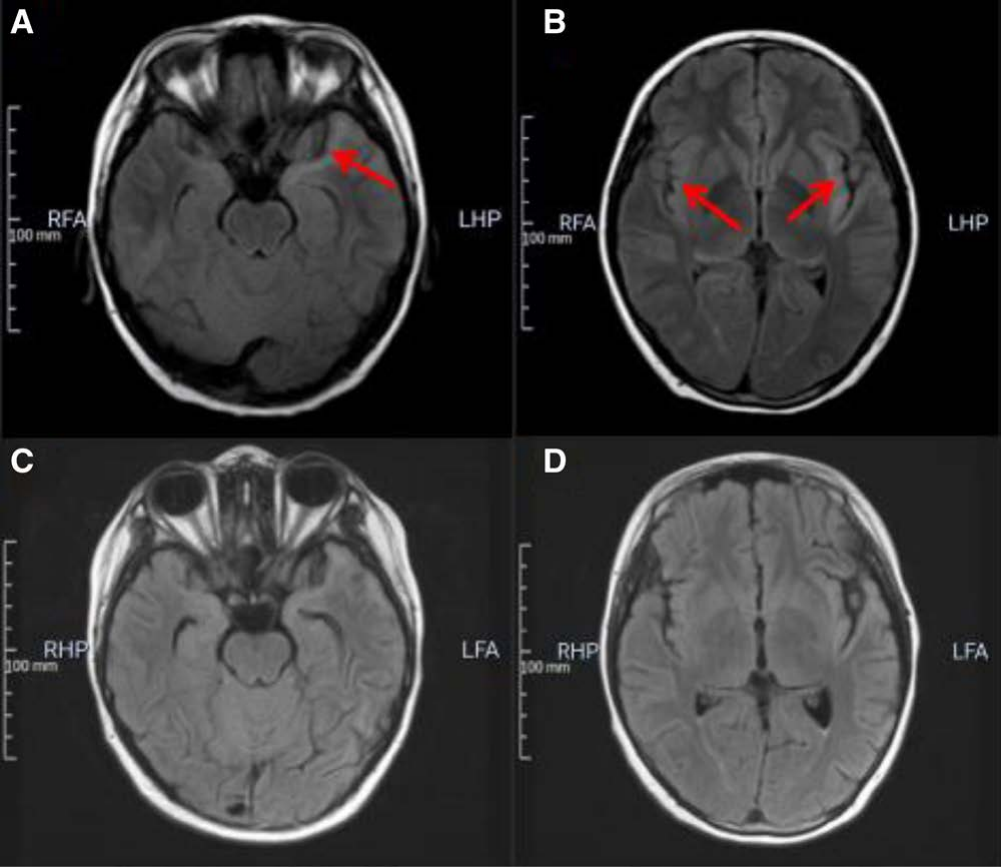
Cerebral MRI in FLAIR image at discharge and 1 yr after discharge. (A and B) showed that hyperintense lesion in the bilateral insular and temporal lobe lesions had no significant changes compared with Fig. 2 (arrowheads). (C and D) showed normal brain tissue 1 yr after discharge. FLAIR = fluid attenuated inversion recovery, MRI = magnetic resonance imaging.

A CSF analysis revealed a white blood cell count of 2 cells/µL and an elevated CSF protein level of 310 mg/dL. The patient was subsequently treated with human immunoglobulin (2 g/kg, divided into 3 doses), mannitol, and a high dose of methylprednisolone succinate (20 mg/kg for 3 consecutive days). The dose of methylprednisolone succinate was then gradually tapered every 3 days until day 18. At this point, the child’s vision in the left eye improved, with visual acuity returning to 0.25. His sensation of taste and pain in the left limb returned to normal, his mental response was good, and tests for antinuclear antibodies were negative. However, there was no significant change in his brain MRI compared to the time of symptom onset, and it did not normalize until 1 year after his discharge from the hospital (Fig. 3).

The child was discharged and prescribed oral prednisone tablets (20 mg, 3 times/d for 6 months). We followed up with him every 6 months for 2 years, during which no recurrence of dizziness, vision loss, taste disturbances or neurological symptoms was observed. His vision gradually improved and returned to normal 8 months after discharge.

## 3. Discussion

The most common diseases that cause dizziness or vertigo are BRV, VM, and benign paroxysmal positional vertigo (BPPV). Patients with BRV and VM typically experience recurrent dizziness or vertigo that can last from a few minutes to several days. Most individuals with these conditions have a family history of headaches, whereas BPPV is a form of temporary vertigo that is induced by moving the head to a specific position for <1 minute. In the present case, the child presented to the otolaryngology department with dizziness following a car trip with his family. His initial physical examination showed no remarkable findings. Based on his history of motion sickness and his score on the MSSQ-short test developed by Golding (2006),^[8]^ he was diagnosed with motion sickness. His dizziness disappeared after 3 days of treatment.

Motion sickness is a common disease in children who attend vertigo clinics.^[9]^ Studies have shown that the incidence of motion sickness in children exceeds 40%,^[10]^ and peaks between the ages of 4 to 12.^[11,12]^ The pathogenesis of motion sickness involves a sensory conflict caused by the difference between current and expected patterns of perceived stimulation.^[3]^ This conflict causes uncomfortable symptoms such as dizziness. Sufficient stimulation can induce motion sickness even in individuals with normal vestibular organ function.^[8]^

Although our patient had a history of dizziness after a car trip, this episode was much longer, and he had no prior history of headaches. As a result, he was initially treated for motion sickness – which is typically a self-limiting disease wherein symptoms resolve within 24 hours of the cessation of vestibular stimulation.^[13]^ The patient’s parents were instructed to observe his condition and immediately return to the doctor if his dizziness did not improve or worsened.

In this case, the symptoms began with dizziness, followed by retrobulbar neuritis, which has not been previously reported. Five cases of MOGAD with dizziness have been reported, all in adults, and all were accompanied by headaches, visual impairment, or gait instability.^[14–18]^ Among these patients, lesions were identified in the brainstem and cerebellum in 2 cases, the right midbrain in 1 case, both temporal lobes in 1 case, and 1 patient had a normal brain MRI. All had optic neuritis. However, retrobulbar neuritis was observed in the case we reported, as the patient had a normal fundus. The existence of taste relies on the perception of taste buds and the neural transmission of taste signals, ultimately reaching the cortical regions of the taste pathway. These cortical regions include the insular (primary), orbitofrontal (secondary), and anterior cingulate (tertiary). In this case, the boy suffered damage to the insular cortex, resulting in a loss of taste sensation.^[19]^ In addition, he was found to have semicircular canal dysfunction, which may be caused by MOGAD or a combination of MOGAD and motion sickness. The vestibular system is composed of 2 parts: the central vestibular system and the peripheral vestibular system. The former refers to the vestibular cortex area, which is the region where some vestibular nerves project to the temporal and parietal lobes of the cerebral cortex through the reticular system. The latter mainly refers to the semicircular canals, utricle and saccule. Lesions in any of these areas may lead to dizziness and impaired vestibular function. In this case, the child may have suffered vestibular dysfunction due to the lesions in the temporal lobe.

Patients with MOGAD typically exhibit either a monophasic or recurrent course. The incidence of the monophasic course is 40% to 50%, and the criterion for both is whether MRI of the brain and spinal cord fully returns to normal.^[20]^ In this case, the boy’s brain MRI returned to normal 1 year after his discharge from the hospital, which indicated that he would likely not experience a relapse of the disease. Although he has not relapsed thus far, at the time of writing this report (two years since his initial presentation), we will continue to follow-up with his family to monitor for any recurrence. Immunotherapy is effective for treating MOGAD in 85% of children,^[21,22]^ with earlier treatment administration correlating with better outcomes and lower recurrence rates. Our patient responded well to immunotherapy and steroid treatment.

## 4. Conclusions

Although most childhood episodes of dizziness are benign, it is important to remain aware of the possibility that they are caused by certain CNS diseases. MOGAD can also cause dizziness in children, not just adults. When the diagnosis is uncertain, it is crucial for doctors and parents to closely monitor affected children to avoid misdiagnoses and treatment delays.

## Acknowledgments

I wish to offer my deep gratitude to everyone who supported me during while doing this research. This includes my colleagues who greatly helped me as well as in my study. Finally, I want to thank my husband and children for their love while making me face life’s challenges and continually pursue my dream.

## Author contributions

**Conceptualization:** Guifang Li, Hua Liang, Yanzhuo Zhang.

**Data curation:** Anqi Zhang, Xinge Lu, Yabo Wang.

**Methodology:** Yabo Wang, Yanzhuo Zhang.

**Resources:** Jinglei Fang.

**Writing – original draft:** Guifang Li, Xinge Lu.

**Writing – review & editing:** Guifang Li, Anqi Zhang, Hua Liang, Jinglei Fang.

## References

[1] Gioacchini FM Alicandri-Ciufelli M Kaleci S Magliulo G Re M. Prevalence and diagnosis of vestibular disorders in children: a review. Int J Pediatr Otorhinolaryngol. 2014;78:718–24. 24612555 10.1016/j.ijporl.2014.02.009

[2] Spinks AB Wasiak J Villanueva EV Bernath V. Scopolamine (hyoscine) for preventing and treating motion sickness. Cochrane Database Syst Rev. 2007;18:CD002851. 10.1002/14651858.CD002851.pub3 17636710

[3] Chang CH Pan WW Tseng LY Stoffregen TA. Postural activity and motion sickness during video game play in children and adults. Exp Brain Res. 2012;217:299–309. 22210118 10.1007/s00221-011-2993-4

[4] Oh J Levy M. Neuromyelitis optica: an antibody-mediated disorder of the central nervous system. Neurol Res Int. 2012;2012:460825. 22363840 10.1155/2012/460825 PMC3272864

[5] Narayan R Simpson A Fritsche K. MOG antibody disease: a review of MOG antibody seropositive neuromyelitis optica spectrum disorder. Mult Scler Relat Disord. 2018;25:66–72. 30048919 10.1016/j.msard.2018.07.025

[6] Whittam DH Karthikeayan V Gibbons E. Treatment of MOG antibody associated disorders: results of an international survey. J Neurol. 2020;267:3565–77. 32623595 10.1007/s00415-020-10026-y PMC7954658

[7] Reindl M Waters P. Myelin oligodendrocyte glycoprotein antibodies in neurological disease. Nat Rev Neurol. 2019;15:89–102. 30559466 10.1038/s41582-018-0112-x

[8] Golding JF. Motion sickness susceptibility. Auton Neurosci. 2006;129:67–76. 16931173 10.1016/j.autneu.2006.07.019

[9] Jahn K. Vertigo and dizziness in children. Handb Clin Neurol. 2016;137:353–63. 27638083 10.1016/B978-0-444-63437-5.00025-X

[10] Henriques IF Douglas de Oliveira DW Oliveira-Ferreira F Andrade PM. Motion sickness prevalence in school children. Eur J Pediatr. 2014;173:1473–82. 24893949 10.1007/s00431-014-2351-1

[11] Bowins B. Motion sickness: a negative reinforcement model. Brain Res Bull. 2010;81:7–11. 19808080 10.1016/j.brainresbull.2009.09.017

[12] Femia P González del Pino B Pérez-Fernández N. Exploración vestibular de niños con alteraciones del equilibrio (I): métodos de la exploración clínica e instrumental [vestibular examination of children with alterations in balance (I): clinical and instrumental examination methods]. Acta Otorrinolaringol Esp. 2011;62:311–7. 21367391 10.1016/j.otorri.2011.01.005

[13] Koch A Cascorbi I Westhofen M Dafotakis M Klapa S Kuhtz-Buschbeck JP. The neurophysiology and treatment of motion sickness. Dtsch Arztebl Int. 2018;115:687–96. 30406755 10.3238/arztebl.2018.0687 PMC6241144

[14] Kojita Y Okada N Hirakawa M Fujii K Satou T Ishii K. Extensive brainstem lesions in myelin oligodendrocyte glycoprotein antibody-associated disease (MOGAD): a case report. Radiol Case Rep. 2024;19:5589–94. 39296754 10.1016/j.radcr.2024.08.032 PMC11406359

[15] Cai MT Lai QL Tang JL. Myelin oligodendrocyte glycoprotein antibody-associated disease preceding primary central nervous system lymphoma: causality or coincidence? Neurol Sci. 2023;44:3711–5. 37389732 10.1007/s10072-023-06919-1

[16] Hong SW Kim BS Park ST Jeong HC Hwang MS Kim SH. General anesthesia, using remimazolam, for the patient with myelin oligodendrocyte glycoprotein antibody associated disease (MOGAD): a case report. Medicine (Baltimore). 2022;101:e31734. 36401433 10.1097/MD.0000000000031734 PMC9678535

[17] Khaladkar SM KirdatPatil PP Dhande A Jhala NA. Myelin oligodendrocyte glycoprotein antibody-associated disease complicated by pachymeningitis: a case report. Cureus. 2024;16:e64868. 39156322 10.7759/cureus.64868 PMC11330373

[18] Anto AM Allu SVV Acharya S Vakde T Omoregi E Pandey U. Uncovering the diagnostic challenge of myelin oligodendrocyte glycoprotein antibody-associated disease: a case study of acute bilateral vision loss. Cureus. 2024;16:e60612. 38903369 10.7759/cureus.60612 PMC11187441

[19] Small DM. Taste representation in the human insula. Brain Struct Funct. 2010;214:551–61. 20512366 10.1007/s00429-010-0266-9

[20] Sechi E Cacciaguerra L Chen JJ. Myelin oligodendrocyte glycoprotein antibody-associated disease (MOGAD): a review of clinical and MRI features, diagnosis, and management. Front Neurol. 2022;13:885218. 35785363 10.3389/fneur.2022.885218 PMC9247462

[21] Waters P Fadda G Woodhall M.; Canadian Pediatric Demyelinating Disease Network. Serial anti-myelin oligodendrocyte glycoprotein antibody analyses and outcomes in children with demyelinating syndromes. JAMA Neurol. 2020;77:82–93. 31545352 10.1001/jamaneurol.2019.2940 PMC6763982

[22] Armangue T Olivé-Cirera G Martínez-Hernandez E.; Spanish Pediatric anti-MOG Study Group. Associations of paediatric demyelinating and encephalitic syndromes with myelin oligodendrocyte glycoprotein antibodies: a multicentre observational study. Lancet Neurol. 2020;19:234–46. 32057303 10.1016/S1474-4422(19)30488-0

